# 657. Evaluating Clinical Outcomes of Bacteremic Patients Treated with AmpC-Inducing Versus -Stable Agents

**DOI:** 10.1093/ofid/ofac492.709

**Published:** 2022-12-15

**Authors:** Barbara Barsoum, Gabriel Karkenny, Henry Donaghy, Thien-Ly Doan

**Affiliations:** Long Island Jewish Medical Center, New Hyde Park, New York; Long Island Jewish Medical Center, New Hyde Park, New York; Northwell Health, New Hyde Park, New York; Long Island Jewish Medical Center, New Hyde Park, New York

## Abstract

**Background:**

AmpC enzymes are β-lactamases that can rapidly hydrolyze penicillins, cephalosporins, and monobactams. When in the presence of β-lactams, inducible pathogens can become derepressed and lead to an overproduction of AmpC. Because of this induction potential, agents that appear susceptible may quickly become resistant, complicating the treatment choices for these infections. The purpose of this study is to assess clinical outcomes in bacteremic patients with an AmpC producing organism based on the antimicrobial agent used.

**Methods:**

This retrospective chart review is approved by Northwell Health IRB and evaluated patients admitted with a positive blood culture from July 2017 to January 2022. The primary objective is outcome of infection (treatment success or failure). Secondary objectives include 30-day readmission related to infection, microbiologic relapse, and in-hospital mortality. Patients were included if they had a positive blood culture demonstrating *E. cloacae*, *E. aerogenes*, or *C. freundii*, cefoxitin resistance, and were treated with a β-lactam antibiotic for at least 72 hours. Patients were excluded if they had polymicrobial cultures or if a resistance gene was detected.

**Results:**

A total of 71 patients were included in analysis, with 40 patients in the carbapenem or cefepime group (Group A), 18 patients in the piperacillin/tazobactam group (Group B), and 13 patients in the ceftriaxone or aztreonam group (Group C). The most frequently isolated pathogen was *E. cloacae* and the most common source of infection was genitourinary. Treatment failure was seen in 16.7% of Group B patients, 15.4% of Group C patients, and 12.5% of Group A patients (p=0.901). Microbiologic relapse was experienced in 5.6% of patients in Group B and 2.5% of patients in Group A (p=0.686). In Group C, 15.4% of patients were readmitted within 30 days related to infection, compared to 11.1% in Group B and 2.5% in the Group A (p=0.23). In-hospital mortality was 16.7% in Group B, compared to 15% in Group A (p=0.341).

**Figure:**
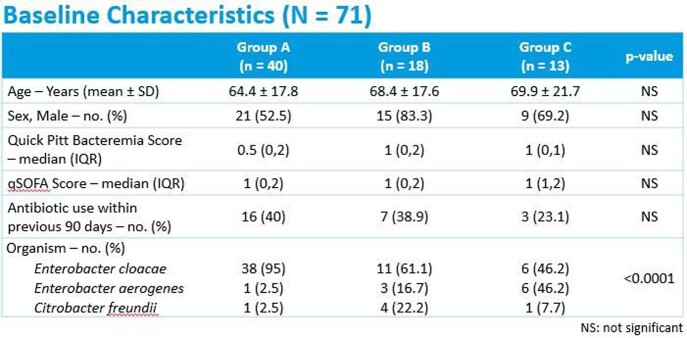

**Conclusion:**

Although not statistically significant, patients who received piperacillin/tazobactam had numerically more treatment failure, microbiologic relapse, and 30-day readmission related to infection when compared to the patients who received a carbapenem or cefepime.

**Disclosures:**

**All Authors**: No reported disclosures.

